# Postpartum depression during the COVID-19 pandemic: an umbrella review and meta-analyses

**DOI:** 10.3389/fpsyt.2024.1393737

**Published:** 2024-07-10

**Authors:** Ali Sahebi, Maryam Kheiry, Kame Abdi, Mahla Qomi, Mohamad Golitaleb

**Affiliations:** ^1^ Department of Medical Emergencies and Health in Disasters and Emergencies, Ilam University of Medical Sciences, Ilam, Iran; ^2^ Non-Communicable Diseases Research Center, Ilam University of Medical Sciences, Ilam, Iraq; ^3^ Nursing Department, Faculty of Medicine, Komar University of Science and Technology, Sulimaniya, Kurdistan Region, Iraq; ^4^ Department of Nursing, Shazand School of Nursing, Arak University of Medical Sciences, Arak, Iran; ^5^ Department of Critical Care Nursing, School of Nursing and Midwifery, Tehran University of Medical Sciences, Tehran, Iran; ^6^ Department of Nursing, School of Nursing, Arak University of Medical Sciences, Arak, Iran

**Keywords:** postpartum depression, COVID-19, systematic review, prevalence, mental disorders

## Abstract

**Introduction:**

The COVID-19 pandemic has significantly increased anxiety, stress, and depression, which could have harmful consequences for pregnant women. Therefore, this study aimed to investigate the prevalence of postpartum depression during COVID-19 using an umbrella review and meta-analysis.

**Methods:**

The current study followed the PRISMA guideline and utilized data from various sources such as PubMed, Scopus, Web of Science, and Google Scholar. The searches were conducted without a time limit until the end of May 2023. Meta-analysis was performed using the random effects model, heterogeneity was assessed using the I2 index, and publication bias was evaluated using Begg’s test. Data analysis was carried out using STATA software (version 15).

**Results:**

In this study, 243 articles were initially identified. Only meta-analysis studies that reported PPD during COVID-19 were included. After quality assessment, nine papers were selected for the meta-analysis stage. The study found that the prevalence postpartum depression (PPD) was 25.27% (95% CI = 23.66–27.86, I^2^ = 0.0%, p = 0.549).

**Conclusion:**

The findings of this study revealed that the incidence of PPD during the COVID-19 pandemic was relatively high. To decrease mental health issues among pregnant and postpartum women, healthcare professionals should implement community programs aimed at preventing, promptly identifying, and providing appropriate intervention for pregnant women. This is crucial as pregnant women are particularly vulnerable to psychological distress during infectious disease outbreaks.

## Introduction

In late 2019, a new corona virus, COVID-19, emerged in China (1, 2). The global spread of the virus led to various crises impacting people’s lives in negative ways. The COVID-19 pandemic significantly increased the prevalence of anxiety, stress, and depression worldwide (3, 4), resulting in serious psychological consequences such as stress, anxiety disorders, depression, and suicidal thoughts (5, 6). Additionally, women were found to be more susceptible to mental health issues compared to men (7, 8), and pregnant women, especially postpartum, were identified as a particularly vulnerable group (9).

After giving birth, women undergo significant hormonal changes that can heavily impact their mental health. These changes may worsen existing psychological conditions or lead to specific disorders such as Postpartum Depression (PPD) (10). This period is recognized as a time of profound depression starting during pregnancy. The prevalence of PPD ranges from 6.5 to 12.9 percent. During the COVID-19 pandemic, it appears that feelings of fear were more widespread (11), and pregnant women experienced heightened levels of anxiety about contracting COVID-19 (12), which was amplified by concerns about the health of the fetus (13).

Due to social distancing and quarantine measures, mothers have experienced limited social support networks and reduced access to healthcare services. This has led to an increased risk of mothers suffering from mental disorders (14, 15).

Micha et al. conducted a study in Greece in 2020 to evaluate the prevalence of postpartum and pregnancy depression and anxiety symptoms during the COVID-19 pandemic. Their findings indicated that high levels of anxiety suggest a need for careful pregnancy monitoring and anxiety screening to identify women who require support during the pandemic. They recommended the implementation of a planned delivery program by healthcare providers to ensure proper antenatal care and postpartum follow-up (16).

It’s important to remember that untreated PPD can have negative consequences for both mothers and babies, impacting pregnancy and fetal development. Psychological disorders such as fear, anxiety, and depression are relatively common in pregnant women who have contracted COVID-19. These disorders can reduce compliance with effective preventive behaviors and lead to unhealthy coping mechanisms that affect the prognosis of pregnancy and fetal development (17).

Research shows that children of mothers with untreated depression are at a higher risk for cognitive disorders, behavioral problems, emotional issues, violent behavior, externalizing disorders, and both psychiatric and medical conditions from birth to adolescence, compared to children of mothers without postpartum depression.

Other studies highlight the specific risks associated with untreated postpartum depression, including weight issues, substance abuse, social difficulties, breastfeeding challenges, and persistent depression (18, 19). Additionally, untreated postpartum depression may lead to an increased risk of suicide and maternal death in the first year after childbirth (20).

There have been numerous systematic reviews and meta-analysis studies on during COVID-19. However, a comprehensive study that reviews these studies and reports the overall prevalence of PPD was not found. The purpose of this study is to report the overall prevalence of PPD during COVID-19, so that the results can be used as a basis for planning and decision-making by managers and policymakers.

## Methods

The study utilized the PRISMA (Preferred Reporting Items for Systematic Reviews and Meta-Analyses) guideline as per reference (21). The study protocol is registered in the International Prospective Register of Systematic Reviews (PROSPERO) under code CRD42023428350.

### Information sources and search strategy

Various information sources, such as Scopus, PubMed, Web of Science, and Google Scholar, were utilized to identify and gather relevant studies. In order to create search strategies, pertinent keywords, search fields, and operators were employed. Initially, a search strategy was formulated for the PubMed database, following which a search strategy was created for the remaining databases based on the PubMed search strategy. The searches were conducted in English with no time constraints up to the end of May 2023. The database search strategies can be found in **Supplementary File** No. 1.

### Eligibility criteria

All studies reporting the prevalence of PPD during the COVID-19 pandemic through meta-analysis were included. Excluded studies were those showing the prevalence of PPD in pregnant women and PPD before the COVID-19 era, as well as review studies that were not meta-analyses.

### Selection of studies

To manage the search results, we first entered all the primary identified studies into EndNote X7 software. Next, we removed duplicate articles and screened the remaining studies. Then, two researchers (AS and MG) independently reviewed the full text of 15 potentially relevant studies. As a result, nine studies were selected for quality assessment.

### Quality assessment and data extraction

Two researchers independently evaluated the quality and methodology of selected studies using the “Multiple Systematic Reviews v2” (AMSTAR-2) tool. They then used a checklist in Word 2016 format to extract data such as the first author, year, sample size, degree of heterogeneity, tools used, and the prevalence rate of PPD in each study. The researchers involved in data extraction were AS and MG.

### Statistical analysis

The included studies were analyzed using a random effects model for a meta-analysis. Index 12 was used to assess the heterogeneity between the studies. Heterogeneity levels were categorized as less than 25%, 25–50%, 50–75%, and more than 75%, indicating no heterogeneity, moderate heterogeneity, high heterogeneity, and very high heterogeneity, respectively (22). Begg’s test was employed to evaluate publication bias, and sensitivity analysis was conducted to determine the impact of each study on the overall prevalence of PPD. The data were analyzed using STATA software (version 15).

## Results

**Figure 1:**
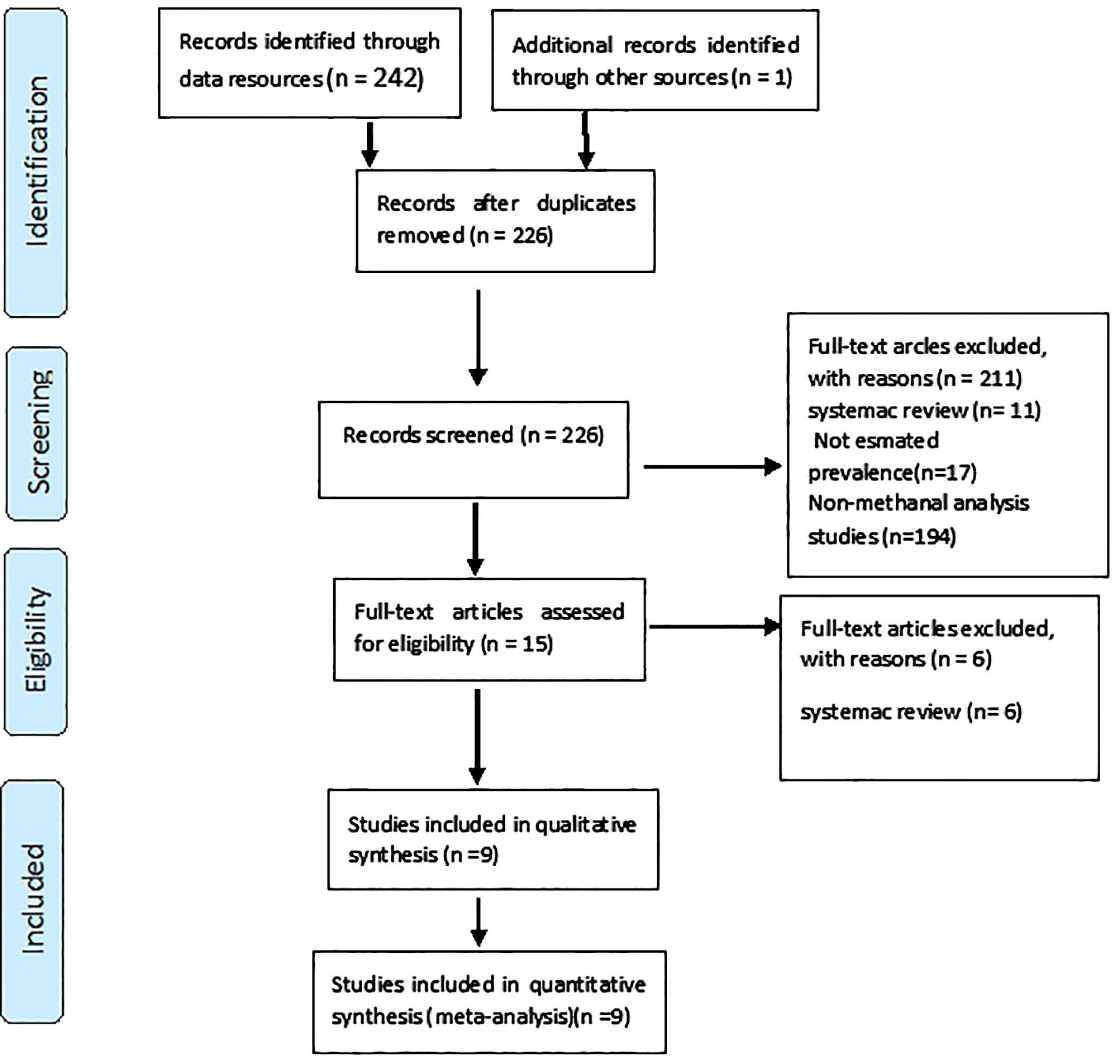
Flowchart of the selection of studies based on PRISMA.

In this study, a total of 243 primary studies were found through a comprehensive search. After removing duplicates, 226 studies were reviewed. Finally, nine studies were qualitatively evaluated and included in the meta-analysis. The process of selecting the studies is depicted in **Figure 1**.

**Figure 2:**
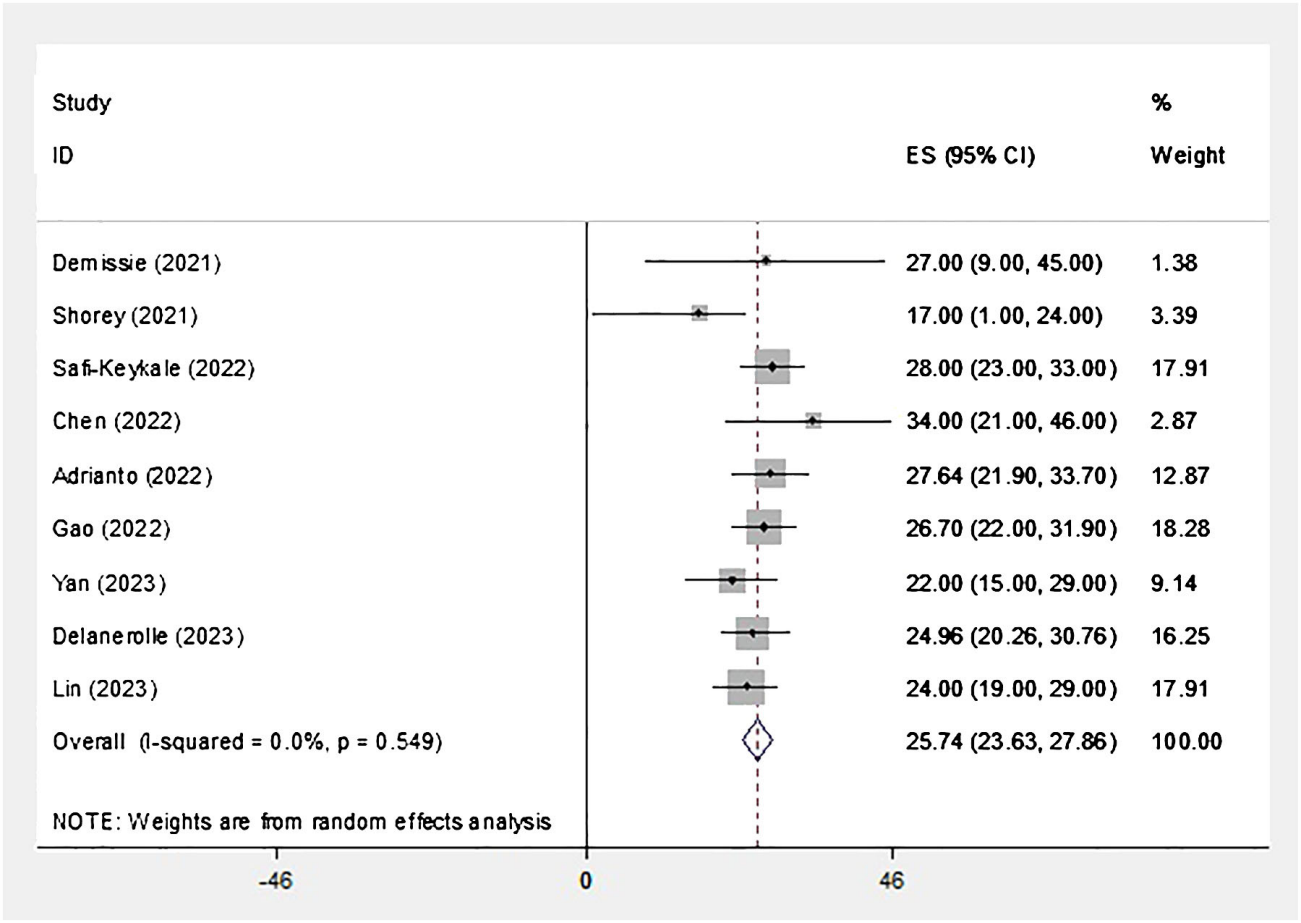
The forest plot of overall and individual prevalence of PPD in the studies with 95% confidence interval.

The specifics of the studies included in the umbrella review are presented in **Table 1**. Moreover, the study reported that the prevalence of postpartum depression (PPD) during COVID-19 was 25.27% (95% CI = 23.66–27.86, I2 = 0.0%, p = 0.549), as shown in **Figure 2**.

**Table 1:** The specifications of studies included in the umbrella review.

First author	Year	N	Prevalence	CI	Heterogeneity	Bias	Tool	Number of included Studies
Adrianto (23)	2022	31497	27.64%	(95% CI, 0.219–0.337)	Q=1132.91, P<0.001, I2=(98.41)	Begg’s test and Egger’s (no publication bias)	PHQ-4 EPDS	19
Chen (24)	2022	6480	34%	(95% CI: 21–46%)	I2 =(73%, p = 0.001)	funnel plot (symmetric)	EPDS	8
Delanerolle (25)	2023	24386	24.96%	(95% CI 20.26–30.76%)	I2=(97.03%)	The p-value of the (Egger’s test was 0.02) for studies of depressive symptoms, revealing the existence of publication bias	EPDS PHQ-9 HADS-D CES-D	29
Demissie (26)	2021	18,335	27%	(95% CI: 9%−45%)	I2=(99.29%, P=0.001)	funnel plot: no publication bias, which was confirmed by an objective test (Egger’s test, P=0.208)	EPDS HADS	14
Gao (27)	2022	20,225	26.7%	(95% CI: 22.0–31.9%)	I2=(98%)	funnel plot and Egger’s test revealed there was no significant publication bias	EPDS PHQ9 CES-D PHQ4	29
Lin (28)	2023		24%	(95% CI: 0.19–0.29)	I2 = (98%)	NR	EPDS PHQ9	26
Safi-Keykaleh (29)	2022	13169	28%	(95% CI = 23–33)	I2 =(98.5%)	Begg’s test (p=0.084)	EPDS PDSS-SF	24
Shorey (30)	2021	4845	17%	(95% CI: 0.10–0.24)	I2=(96%)	funnel plots, the asymmetry indicates potential publication bias.	EPDS	5
Yan (31)	2022	3,759	22%	(95% CI 15–29%)	I2 = (85.7%)	NR	EPDS	3

N, Number; CI, Confidence interval; EPDS, Edinburgh Postnatal Depression Scale (EPDS); PDSS-SF, Postpartum Depression Screening Scale-Short Form; PHQ-4, Patient Health Questionnaire-4; PHQ-9, Patient Health Questionnaire-9; HADS-D, Hospital Anxiety and Depression Scale; CES-D, Center for Epidemiological Studies Depression; NR, Not reported.

**Figure 3:**
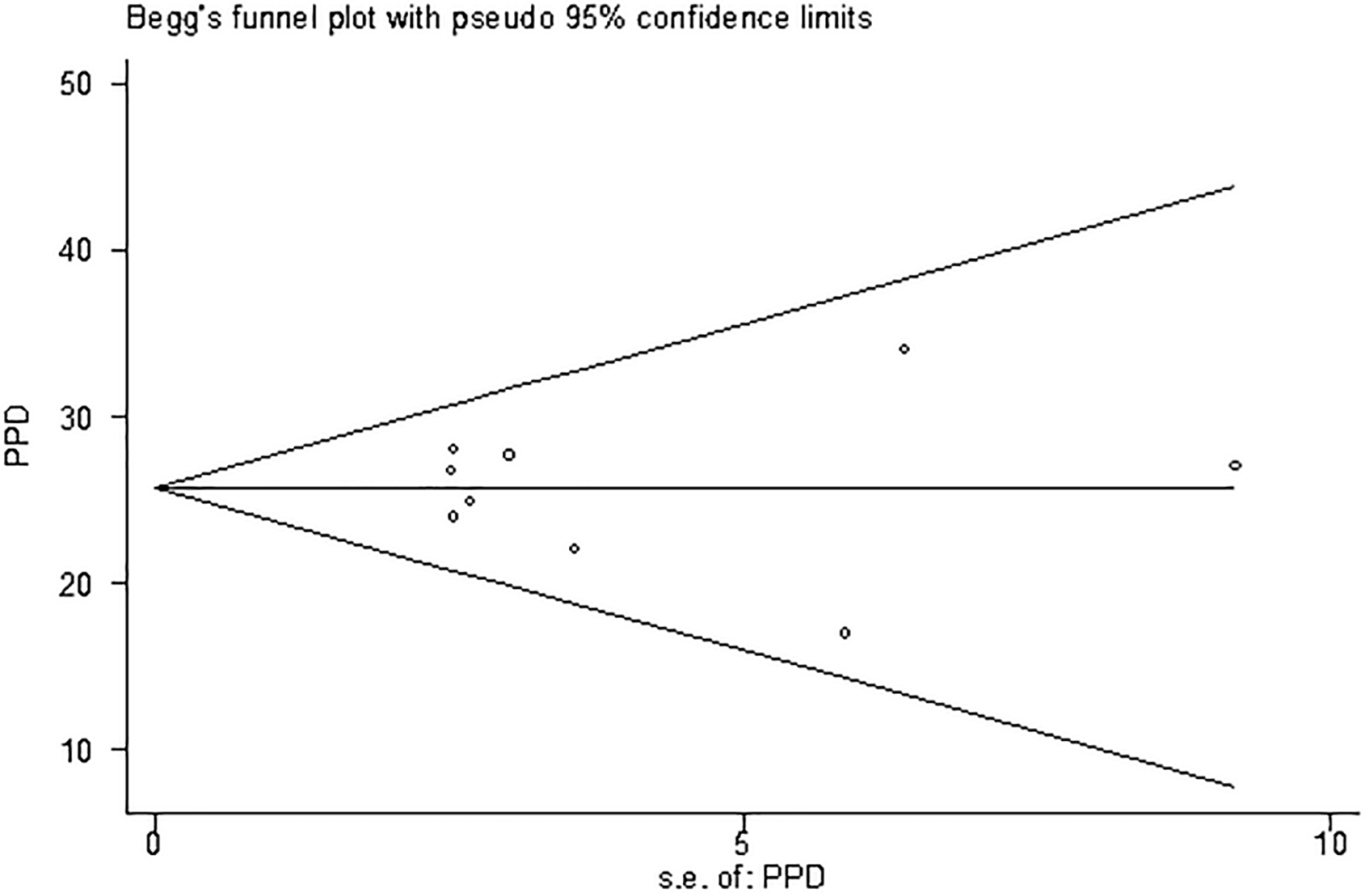
Publication bias based on Begg test.

**Figure 4:**
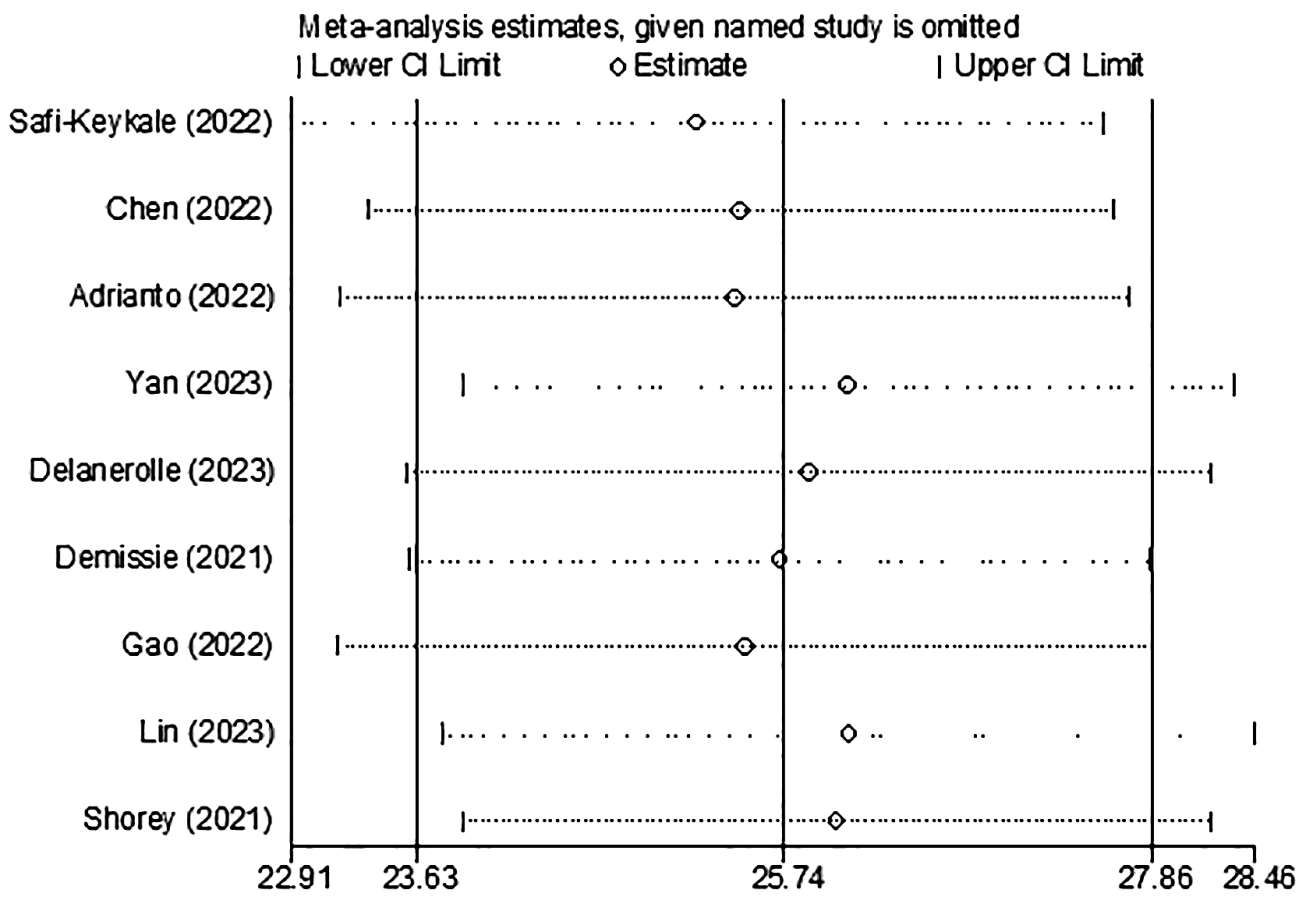
Sensitivity analysis for the prevalence of PPD during COVID-19.

The I2 index indicates that there is no heterogeneity among the studies. Based on the results of Begg’s test (p = 0.753), there is no significant publication bias, as shown in **Figure 3**. The sensitivity analysis revealed that the prevalence of PPD remained unchanged even after removing each study individually (**Figure 4**).

## Discussion

The review found that PPD during the COVID-19 pandemic was 25.27%. Nine studies that met the inclusion criteria were evaluated, and all of them used the EPDS to assess depressive symptoms. based on the I^2^ index, it was shown that there is no heterogeneity among studies.

Gao’s study specifically showed that the incidence of PPD during the COVID-19 pandemic was 26.7%. It also found that women under 35 years old, with low income, lower education, and not breastfeeding were at a higher risk of postpartum depression or anxiety symptoms at 6 weeks postpartum (27). According to research by Wang et al., the global incidence of postpartum depression (PPD) is 17.22% (32).

Another review study by Liu et al. found that PPD affects around 14% of women, with the rates varying from 5% to 26.83% depending on the country. The incidence of PPD tends to be higher in developed countries. Risk factors for developing PPD include diabetes mellitus, epidural anesthesia during childbirth, a history of depression during or before childbirth, and giving birth to a male baby (33).

Studies have indicated that COVID-19 plays a significant role in causing depression during pregnancy and postpartum (29). The COVID-19 outbreak has significantly affected vulnerable groups, particularly pregnant women. The period during and after pregnancy is a vulnerable time that can lead to increased distress for many women (34).

In countries like Iran, PPD is prevalent and raising awareness among families about the importance of adequate social support for mothers may help prevent the occurrence of this disorder (35). Factors such as premature birth, the baby’s sex, the mother’s illness, stressful events like family member deaths, low social status, and current hospitalization of a child can all influence the development of PPD (36, 37).

The prevalence of this disorder increases before childbirth, and gradually decreases after the birth of the child, and is more common in primiparous women than multiparous ones (38). Various studies have shown that telephone and pharmaceutical nursing interventions can be effective in the treatment of PPD, and perinatal counseling interventions can be effective in their prevention (39, 40).

Research has found that the prevalence of PPD was higher during the COVID-19 pandemic in mothers who used expressed breast milk and complementary feeding for their children, those with little social support, mothers who consumed insufficient or low-nutritional food during pregnancy and childbirth, and those who had experienced health problems. Additionally, PPD was more common among women aged 18 to 29 and less prevalent in women who had a planned pregnancy (41). The prevalence of PPD was also higher in mothers who smoked and were admitted to the hospital, especially the ICU, during the COVID-19 pandemic (42).

In some Asian countries such as India, Vietnam, China, and Turkey, as well as in some African countries like Uganda and Egypt, the birth of a female child is considered a risk factor for postpartum depression (PPD). This is due to the cultural expectation that sons will provide financial support for the family (43–45). In higher-income Asian countries, factors such as family and marital conflicts, lack of social support, intimate partner violence, and the stress of raising multiple children due to financial concerns can contribute to the increasing prevalence of PPD (46).

During the COVID-19 pandemic, immigrant women with persistent fever, limited social support, and anxiety about contracting COVID-19 were found to be at a higher risk of PPD. Conversely, women who did not share utensils and tools during the pandemic were at a lower risk of infection (47). Another study suggested that factors such as advanced maternal age, childcare concerns, living conditions, lack of social support, dissatisfaction with marital relationships, and unintended pregnancy may contribute to the occurrence of postpartum depression during the COVID-19 pandemic (48).

The prevalence of PPD was found to be high among older, single, unemployed women, as well as women who lost their jobs or were dissatisfied with their household income due to the pandemic. Additionally, PPD was significantly associated with factors such as quarantine, social isolation, lack of social support, and emotional distress (49).

Factors contributing to the level of depression in pregnant women during the COVID-19 pandemic era based on previous studies include: mother’s and partner’s occupation, mother’s and partner’s education level, social support, parity, consumption of comfort food, marital satisfaction, use of social media, high-risk pregnancy, sleep patterns, concerns about the transmission of COVID-19 to mothers and babies, worries about COVID-19, light exercise, and limited access to health services (50).

The COVID-19 pandemic has led to restrictions on meeting and visiting relatives, as well as limitations on hospital visits and postpartum care by medical staff. These measures, aimed at minimizing contact to prevent the spread of COVID-19, have also contributed to an increase in the prevalence of PPD.

Comparing the results of the current study with previous studies, we can conclude that the prevalence of PPD is high globally, especially in developed countries and during the COVID-19 pandemic. This not only affects the mother’s health but also the baby’s health and that of other family members. During COVID-19, in addition to experiencing significant events such as pregnancy and childbirth, mothers are exposed to severe psychological consequences, especially stress and anxiety, which can increase the risk of PPD.

To prevent or reduce PPD, it is recommended that mothers, as a vulnerable group, receive special physical and psychological care and support during the COVID-19 era. In cases of depression symptoms, more specific interventions are necessary. Frequent follow-ups by health centers and hospitals should also be done because PPD may affect mothers’ social behaviors and lead to persistent depression. Therefore, health care providers should screen mothers for depression during the COVID-19 pandemic, from early pregnancy through the postpartum period.

### Limitations

The nature of this study did not allow for subgroup analysis based on the type of tool used. As umbrella studies are tertiary studies, conducting subgroup analysis was not possible due to the limited reporting of variables in primary and secondary studies. Additionally, because our study is based on meta-analysis articles published on PPD during the COVID-19 pandemic in different countries, subgrouping analysis based on variables such as country of residence, social class, maternal and paternal occupation and education, social support, parity, comfort eating, marital satisfaction, and use of social media was not feasible.

### Suggestions for future studies

It is recommended to examine and compare depression in women at different stages such as before pregnancy, during pregnancy, and breastfeeding, especially during the COVID-19 pandemic.

## Conclusion

The results of this umbrella review revealed that the prevalence of PPD among women is high globally, especially in developed countries, and as a result, it can have a significant impact on the health of the mother and the child. Due to the high prevalence of postpartum depression, the focus is on prevention nowadays, so it is necessary to take preventive measures to minimize PPD.

The postpartum period is a challenging time for new mothers, so it’s important to provide emotional and psychological support in both community and healthcare settings to reduce the risk of depression. Postpartum depression can lead to general health issues. Many of the factors identified in this review can be addressed through a combination of maternal and neonatal healthcare. Thus, it’s crucial to consider screening programs and policies, as well as strengthen postpartum care programs, to minimize postpartum depression.

## Data availability statement

The original contributions presented in the study are included in the article/**Supplementary Material**. Further inquiries can be directed to the corresponding author.

## Author contributions

AS: Conceptualization, Investigation, Methodology, Resources, Visualization, Writing – original draft. MK: Methodology, Writing – original draft. KA: Resources, Validation, Writing – review & editing. MQ: Investigation, Software, Writing – original draft. MG: Conceptualization, Data curation, Investigation, Software, Supervision, Validation, Writing – review & editing.
