# Supplementary material for: Postpartum depression during the COVID-19 pandemic: an umbrella review and meta-analyses
Source: Front Psychiatry. 2024 Jul 10;15:1393737. doi: 10.3389/fpsyt.2024.1393737 (PMC11266160; doi:10.3389/fpsyt.2024.1393737)
Supplement: Supplementary file 1 [file Table_1.docx]

Supplementary files 1. Search syntax for types database

| Databases | Search Syntax | N |
| --- | --- | --- |
| PubMed | ((“Postnatal Depression” OR “Post-Partum Depression” OR “Postpartum Depression” OR “Post-Natal Depression” OR “Post Natal Depression” OR “Postnatal Dysphoria” OR “Post-Partum Dysphoria” OR “Post Partum Dysphoria” OR “Postpartum Dysphoria” OR “Post-Natal Dysphoria” OR “Post Natal Dysphoria”) AND (“COVID 19” OR “2019-nCoV Infection*” OR “2019 nCoV Infection” OR “SARS-CoV-2 Infection” OR “SARS CoV 2 Infection*” OR “2019 Novel Coronavirus Disease” OR “2019 Novel Coronavirus Infection” OR “COVID-19 Virus Infection” OR “COVID-19 Virus Infection*” OR COVID19 OR “Coronavirus Disease 2019” OR “Coronavirus Disease-19” OR “Coronavirus Disease 19” OR “Severe Acute Respiratory Syndrome Coronavirus 2 Infection” OR “COVID-19 Virus Disease*” OR “SARS Coronavirus 2 Infection” OR “2019-nCoV Disease*” OR “COVID-19 Pandemic*” OR “COVID 19 Pandemic”) AND (“Systematic review” OR “meta-analysis” OR “meta-analytic”)) | 14 |
| Scopus | ((ALL(“Postnatal Depression”) OR ALL(“Post-Partum Depression”) OR ALL(“Postpartum Depression”) OR ALL(“Post-Natal Depression”) OR ALL(“Post Natal Depression”) OR ALL(“Postnatal Dysphoria”) OR ALL(“Post-Partum Dysphoria”) OR ALL(“Post Partum Dysphoria”) OR ALL(“Postpartum Dysphoria”) OR ALL(“Post-Natal Dysphoria”) OR ALL(“Post Natal Dysphoria”)) AND (ALL(“COVID 19”) OR ALL(“2019-nCoV Infection*”) OR ALL(“2019 nCoV Infection”) OR ALL(“SARS-CoV-2 Infection”) OR ALL(“SARS CoV 2 Infection*”) OR ALL(“2019 Novel Coronavirus Disease”) OR ALL(“2019 Novel Coronavirus Infection”) OR ALL(“COVID-19 Virus Infection”) OR ALL(“COVID-19 Virus Infection*”) OR ALL(COVID19) OR ALL(“Coronavirus Disease 2019”) OR ALL( “Coronavirus Disease-19”) OR ALL(“Coronavirus Disease 19”) OR ALL(“Severe Acute Respiratory Syndrome Coronavirus 2 Infection”) OR ALL( “COVID-19 Virus Disease*”) OR ALL(“SARS Coronavirus 2 Infection”) OR ALL(“2019-nCoV Disease*”) OR ALL(“COVID-19 Pandemic*”) OR ALL(“COVID 19 Pandemic”)) AND (TITLE-ABS (“Systematic review”) OR TITLE-ABS (“meta-analysis”) OR TITLE-ABS( “meta-analytic”))) | 218 |
| ISI | ((TS=(“Postnatal Depression”) OR TS=(“Post-Partum Depression”) OR TS=(“Postpartum Depression”) OR TS=(“Post-Natal Depression”) OR TS=(“Post Natal Depression”) OR TS=(“Postnatal Dysphoria”) OR TS=(“Post-Partum Dysphoria”) OR TS=(“Post Partum Dysphoria”) OR TS=(“Postpartum Dysphoria”) OR TS=(“Post-Natal Dysphoria”) OR TS=(“Post Natal Dysphoria”)) AND (TS=(“COVID 19”) OR TS=(“2019-nCoV Infection*”) OR TS=(“2019 nCoV Infection”) OR TS=(“SARS-CoV-2 Infection”) OR TS=(“SARS CoV 2 Infection*”) OR TS=(“2019 Novel Coronavirus Disease”) OR TS=(“2019 Novel Coronavirus Infection”) OR TS=(“COVID-19 Virus Infection”) OR TS=(“COVID-19 Virus Infection*”) OR TS=(COVID19) OR TS=(“Coronavirus Disease 2019”) OR TS=( “Coronavirus Disease-19”) OR TS=(“Coronavirus Disease 19”) OR TS=(“Severe Acute Respiratory Syndrome Coronavirus 2 Infection”) OR TS=( “COVID-19 Virus Disease*”) OR TS=(“SARS Coronavirus 2 Infection”) OR TS=(“2019-nCoV Disease*”) OR TS=(“COVID-19 Pandemic*”) OR TS=(“COVID 19 Pandemic”)) AND (TS=(“Systematic review”) OR TS=(“meta-analysis”) OR TS=( “meta-analytic”))) | 11 |
|  |  |  |
